# First mesomorphic and DFT characterizations for 3- (or 4-) *n*-alkanoyloxy benzoic acids and their optical applications

**DOI:** 10.1016/j.heliyon.2023.e19384

**Published:** 2023-08-24

**Authors:** Mohamed A. El-Atawy, Mohd Taukeer Khan, Saheed A. Popoola, Muna S. Khushaim, Mariusz Jaremko, Abdul-Hamid Emwas, Fowzia S. Alamro, Magdi M. Naoum, Hoda A. Ahmed

**Affiliations:** a Chemistry Department, Faculty of Science, Alexandria University, P.O. Box 426, Ibrahemia, Alexandria 21321, Egypt; b Chemistry Department, College of Sciences, Taibah University, Yanbu 30799, Saudi Arabia; c Department of Physics, Faculty of Science, Islamic University of Madinah, Al-Madinah Al-Munawwarah 42351, Saudi Arabia; d Chemistry Department, Faculty of Science, Islamic University of Madinah, Madinah 42351, Saudi Arabia; e Department of Physics, Faculty of Science, Taibah University, P.O. Box 30002, Al-Madina 41447, Saudi Arabia; f Strategic Research Labs, Taibah University, P.O. Box 30002, Al-Madina 41447, Saudi Arabia; g Biological and Environmental Sciences & Engineering Division (BESE), King Abdullah University of Science and Technology (KAUST), Thuwal, 23955-6900, Saudi Arabia; h Core Labs., King Abdullah University of Science and Technology, Thuwal 23955-6900, Saudi Arabia; i Department of Chemistry, College of Science, Princess Nourah Bint Abdulrahman University, P.O. Box 84428, Riyadh 11671, Saudi Arabia; j Department of Chemistry, Faculty of Science, Cairo University, Giza 12613, Egypt

**Keywords:** 3- (or 4-)-*n*-alkanoyloxy benzoic acids, Symmetrical supramolecular-H bonding, Smectic phase, Characterizations, Mesophase stability, DFT, Optical properties

## Abstract

New liquid crystalline hydrogen bonded 3- (or 4)-*n*-alkanoyloxy benzoic acids were synthesized and probed theoretically and experimentally. The molecular structures of these compounds were elucidated by proton NMR, carbon-13 NMR and elemental analyses. Differential scanning calorimetry (DSC) was used to investigate the thermal and mesomorphic properties of all the symmetrical dimers that bearing identical alkanoyloxy chains. Moreover, polarized optical microscopy (POM) was used to determine their mesophases. The findings show that all the designed symmetrical dimers exhibit the smectic mesophase with relative thermal stability that depends on the length of their terminal side chain. Additionally, the experimental findings of the mesomorphic behavior are further supported by DFT calculations. The alkanoyloxy benzoic acid para-derivatives (**I**n) were shown to be more stable than their meta-substituted (**II**n) analogues due to stronger hydrogen bonding interactions. The computed reactivity parameters showed that the position of ester moiety has a significant impact on the acids reactivity. The absorbance spectra of both the 3- (or 4)-*n*-alkanoyloxy benzoic acids revealed a blue shift with the increment of the of alkyl chain size; however, the energy band gaps of 3-*n*-alkanoyloxy benzoic derivatives were found to be slightly higher than those of the 4-*n*-alkanoyloxy benzoic acids. Moreover, the photoluminescence spectrum of the prepared materials is rather broad, and exhibited a red shift as the alkyl chain length increases. The fluorescence lifetime shown to rise as alkyl chain length grows longer, and 3-*n*-alkanoyloxy benzoic acids have slightly longer lifetime compared to their 4-*n*-alkanoyloxy benzoic analogues.

## Highlights

• First mesomorphic and DFT characterizations for *n*-alkanoyloxy benzoic acids.
• Experimental and computational investigations were carried out for optical applications.
• Optical applications and electrical properties were conducted.
• Increasing the length of the alkanoyl side chain was shown to enhance the optical bandgap.
• Experimental behaviors offer the designed complexes suitable for energy applications.

## 1 Introduction

Owing to promising mesomorphic and optical properties, the liquid crystals (LCs) materials were shown to be useful in many different fields, such as those of screens, solar cells, sensors, and modulators [1]. The fields of biology, agriculture, medicine, and oil recovery are all good fits for LCs. Consequently, there has been an uptick in recent efforts to study, develop, and create novel liquid crystalline structures [[2], [3], [4], [5]]. In recent years, a lot of interest has been devoted to supramolecular interactions originating from Hydrogen-bonded systems [[6], [7], [8], [9], [10], [11]]. Additionally, non-covalently interacting LC materials exhibited a variety of applications. Moreover, Supramolecular LC complexes (SMLCs) include molecular association and are crucial in many biochemical and biological processes. The molecular architecture is the defining factor in determining the shape of the LC molecules. [[12], [13], [14]], and hence contribute significantly in the formation of mesophase. Mesomorphic stability is mostly determined by the polarizability and/or polarity of the molecular core mesogens. Calamitic intermolecular H-bonding interactions were included within the core of many symmetrical and non-symmetrical H-bonded mesogens [[15], [16], [17], [18], [19], [20]]. The formation of angular complexes by intermolecular hydrogen bonding between molecules was the subject of another investigation [21]. It has also been revealed that mesogenic derivatives play a crucial role as a linking group in the formation of LC dimers [[22], [23], [24], [25], [26]].

In the recent years, hydrogen bonding interaction has been recognized in the production and stability of LC mesophases [27], and its involvement in mesophase self-assembly has also been studied [28,29]. Accordingly, in thermotropic LC systems, intermolecular hydrogen bonding interactions have shown promising potential applications [30,31]. Furthermore, aromatic carboxylic acid dimerization is the first example of LC production [32]. The geometry of the resulting SMLCs has a significant impact on the behavior of LC material. Pyridyl and carboxylic components also serve as proton-accepting and proton-donating moieties, respectively, in several SMLCs [33,34].

Gray et al. [35] presented the earliest known examples of systems in which hydrogen bonds between molecules created a liquid crystal. Due to their mesomorphic properties, alkoxy-substituted benzoic acids produce calamitic symmetric mixtures of interacting benzoic acid molecules that form supramolecular hydrogen bonding (SMHB) interactions [35,36]. This strategy has recently been expanded to the development of novel supramolecular complex such as new twist-bent nematogens (bent SMHBLCs) [37]. In general, hydrogen bonding is more effective than covalent bonding for producing supramolecular mesogens because they have extended mesogenic part. Moreover, a new method for incorporation of functionality into the molecular skeleton in a controlled and effective manner has been developed [38].

Usually, not a single compound achieves all the desired fundamental characteristics; hence, most LC display materials used in devices today are hybrids. This has made the study of LC component mixtures are major topics [37]. The derivatives of benzoic acid are the most commonly used compounds for the development of the LC materials via hydrogen bonding interactions [38].

Liquid crystals may be formed through H-bonding interactions involving the benzoic acid derivatives [38]. Their ability to form ordered structures through intermolecular hydrogen bonding makes them suitable for various technological applications. With ongoing research and development, the field of LCs continues to expand, opening up new possibilities for their utilization in advanced electronic and optical devices. The formation of liquid crystals typically involves molecular self-assembly, where the molecules organize themselves into ordered structures due to interactions between molecules such as hydrogen bonding, dipole-dipole interactions, and van der Waals forces. In the case of benzoic acid derivatives, the development of liquid crystals is greatly influenced by hydrogen bonding. The ability of benzoic acid derivatives to form liquid crystals via H-bonding is crucial for their application in various technological devices. Liquid crystals are distinguished by their dual properties as both a liquid and a solid. , making them highly suitable for applications requiring both mobility and organization. By carefully selecting the substituent groups in benzoic acid derivatives and controlling the conditions, such as temperature and pressure, researchers can manipulate the formation and properties of LCs. For example, by introducing bulky substituents, the distance between the molecules increases, leading to a decrease in intermolecular interactions and a change in the liquid crystal phase. This flexibility in tailoring the characteristics of LCs allows for their customization to suit specific device requirements [[39], [40], [41], [42]].

Computational studies for liquid crystals provide a deep understanding of their complex behavior and enable the design of advanced materials for various applications [[43], [44], [45]]. These studies contribute to the advancement of liquid crystal technologies, leading to improved displays, sensors, and other optoelectronic devices. Computational studies often start with the development of molecular models that accurately represent the structure and interactions of liquid crystals. Force field parameters are assigned to describe intermolecular forces, such as electrostatic and van der Waals interactions. These models aim to capture the complex behavior of liquid crystals, including phase transitions and the formation of ordered mesophases. One of the primary focuses of computational studies is to understand the phase behavior of LCs. By simulating the behavior of different molecules under varying temperatures and pressures, researchers can predict the occurrence of phase transitions, such as isotropic to nematic, nematic to smectic, and smectic to crystalline transitions. This information is invaluable for designing and optimizing LC-based materials for various applications, including sensors, displays, and photonic devices. Computational studies also seek to understand the self-assembly and organization processes in liquid crystals. Molecules in a liquid crystal material can form various ordered structures, such as layers, columns, and helical arrangements. Simulations provide insights into the factors that influence the formation of these structures, including molecular shape, size, and intermolecular interactions. By tuning these factors, researchers can control and engineer the desired properties of liquid crystals. LCs are widely employed in display technologies due to their ability to respond to external stimuli, such as electric fields. Computational studies play an important role in estimating and understanding the optoelectronic properties of liquid crystals, including their optical anisotropy, refractive index, and electro-optic response. This information guides the creation of novel liquid crystal-based devices, such as liquid crystal displays (LCDs) and optical switches. Computational studies act as a powerful tool in the design and discovery of new LC materials. By screening large databases of molecular structures and employing computer algorithms, researchers can identify promising candidates with desired properties. Once potential materials are identified, simulations help in understanding their behavior, stability, and performance before experimental synthesis and characterization.

Small changes in bonding can have large effects on geometrical arrangement of atoms and vibrational frequencies, which must be taken into account when studying a novel class of the mesogenic moieties as well as the flexible spacers in the supramolecular complexes. The goal of the present study is to examine the mesomorphic behavior of the possible SMLC formation via hydrogen bonding interactions (**I***n* and **II***n*) produced between pairs of 3- (or 4-)-*n*-alkanoyloxy benzoic acid derivatives with varying terminal alkanoyloxy chain lengths. The influence of the mesogenic cores as a function of the location of the alkanoyloxy chain in the designed complexes was also investigated. Moreover, the experimental results wasuld be substantiated by the theoretical calculations via DFT approach. The DFT-based quantum computation of **I***n* and **II***n* were performed to investigate the important electrical parameters including position of E_HOMO_ and E_LUMO_, energy gap, dipole moments, ionization potential, electron affinity [46,47]. Moreover, the molecular structural and thermodynamical parameters were also evaluated from the DFT calculation. The performance of an optoelectronic devices is greatly influenced by these parameters [48], therefore, the knowledge of these parameters is vital to develope efficient optoelectrical devices. Further, in order to find the application of prepared LC materials in nematic display devices, the optical and photophysical properties were investigated by recording the absorption spectra, steady state and time resolved spectra and important electrical parameters such energy band gap and charge carriers lifetime were evaluated.

## 2 Experimental

### 2.1 Synthesis

**Scheme 1:**
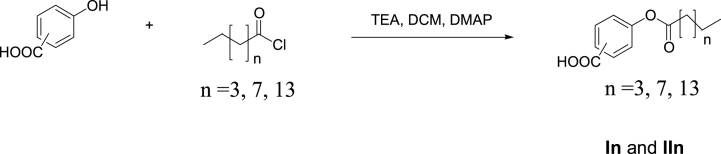
Synthesis of title groups, **I***n* and **II***n*.

In order to produced alkanoyloxy benzoic acid derivatives (In and IIn) hydroxybenzoic acid (20 mmol) in 40 mL of DCM, Triethylamine TEA (40 mmol), dimethylaminopyridine (DMAP) (2 mmol) and the appropriate acid chloride (20 mmol) were mixed at zero degrees Celsius. The reaction mixture was stirred and kept at room temperature overnight. The volatiles were removed under reduced pressure, and the resultant oily residue was then dissolved in aqueous solution of 1 N HCl and extracted with diethylether (5 × 20 mL). After washing with NaHCO_3_, drying over Na_2_SO_4_, filtering, and concentrating under low pressure, we obtained the final organic layer. Finally, the purification was carried out by the crystallization from ethyl alcohol, which yields the desired esters as illustrated in Scheme 1.

#### 2.1.1 4-(Hexanoyloxy) benzoic acid

^1^H NMR (850 MHz, DMSO) *δ* 7.99 (d, *J* = 8.5 Hz, 2H, Ar–H), 7.25 (d, *J* = 8.6 Hz, 2H, Ar–H), 2.60 (t, *J* = 7.4 Hz, 2H, CH_2_), 1.67–1.63 (m, 2H, CH_2_), 1.36–1.32 (m, 4H, 2CH_2_), 0.89 (t, *J* = 7.1 Hz, 3H, CH_3_). ^13^C NMR (214 MHz, DMSO) *δ* 171.96 (CO), 167.12 (CO), 154.49 (C), 131.60 (CH), 128.75 (C), 122.48 (CH), 33.89 (CH_2_), 31.04 (CH_2_), 24.40 (CH_2_), 22.26 (CH_2_), 14.26 (CH_3_).

#### 2.1.2 Decanoic 4-(decanoyloxy) benzoic anhydride

^1^H NMR (850 MHz, DMSO) *δ* 7.99 (d, *J* = 8.3 Hz, 2H, Ar–H), 7.23 (d, *J* = 8.4 Hz, 2H, Ar–H), 2.59 (t, *J* = 7.4 Hz, 2H, CH_2_), 2.18 (t, *J* = 7.4 Hz, 2H, CH_2_), 1.67–1.62 (m, 2H, CH_2_), 1.49 (m, 2H, CH_2_), 1.26 (m, 22H, 11CH_2_), 0.86 (t, *J* = 6.9 Hz, 6H, 2CH_3_)·^13^C NMR (214 MHz, DMSO) *δ* 174.75 (CO), 171.93 (CO), 167.18 (CO), 154.29, 131.30, 128.46, 122.39, 49.05, 34.14, 33.93, 33.72, 31.75, 29.35, 29.31, 29.23, 29.17, 29.13, 29.11, 29.02, 28.92, 28.84, 24.97, 24.72, 22.56, 14.39.

#### 2.1.3 4-(Palmitoyloxy) benzoic acid

^1^H NMR (850 MHz, DMSO) *δ* 7.99 (d, *J* = 8.6 Hz, 2H, Ar–H), 7.24 (d, *J* = 8.6 Hz, 2H, Ar–H), 2.60 (t, *J* = 7.4 Hz, 2H, CH_2_), 1.67–1.62 (m, 2H, CH_2_), 1.38–1.33 (m, 2H, CH_2_), 1.31–1.20 (m, 22H, 11CH_2_), 0.86 (t, *J* = 7.1 Hz, 3H, CH_3_). ^13^C NMR (214 MHz, DMSO) *δ* 171.89 (CO), 167.17 (CO), 154.25 (C), 131.25 (CH), 128.75 (C), 122.48 (CH), 33.93 (CH_2_), 31.76 (CH_2_), 29.52 (CH_2_), 29.50 (CH_2_), 29.47 (CH_2_), 29.47 (CH_2_), 29.41 (CH_2_), 29.30 (CH_2_), 29.17 (CH_2_), 29.11 (CH_2_), 28.80 (CH_2_), 24.71 (CH_2_), 22.56 (CH_2_), 14.42 (CH_3_).

#### 2.1.4 3-(Decanoyloxy) benzoic acid

^1^H NMR (400 MHz, CDCl_3_) *δ* 8.82 (brs, 1H, OH), 7.96 (d, *J* = 7.4 Hz, 1H, Ar–H), 7.80 (s, 1H, Ar–H), 7.46 (t, *J* = 7.7 Hz, 1H, Ar–H), 7.29 (d, *J* = 7.6 Hz, 1H, Ar–H), 3.18 (m, 2H, CH_2_CO), 2.47–1.36 (m, 14H, 7CH_2_), 0.90 (s, 3H, CH_3_)·^13^C NMR (101 MHz, CDCl_3_) *δ* 179.91 (CO), 172.31 (CO), 150.52 (C), 132.30 (C), 129.33 (CH), 127.34 (CH), 126.43 (CH), 123.24 (CH), 45.51 (CH_2_), 34.33 (CH_2_), 31.86 (CH_2_), 29.41 (CH_2_), 29.26 (CH_2_), 29.10 (CH_2_), 24.89 (CH_2_), 22.66 (CH_2_), 14.09 (CH_3_).

#### 2.1.5 3-(Palmitoyloxy) benzoic acid

^1^H NMR (400 MHz, DMSO) *δ* 7.84 (d, *J* = 7.7 Hz, 1H, Ar–H), 7.64 (s, 1H, Ar–H), 7.56 (t, *J* = 7.9 Hz, 1H, Ar–H), 7.38 (d, *J* = 8.1 Hz, 1H, Ar–H), 2.60 (t, *J* = 7.3 Hz, 2H, CH_2_CO), 1.74–1.57 (m, 2H, CH_2_), 1.28 (d, *J* = 32.5 Hz, 14H, 7CH_2_), 0.85 (t, *J* = 6.6 Hz, 3H, CH_3_). ^13^C NMR (101 MHz, DMSO) *δ* 172.07(CO), 168.43 (CO), 150.97 (C), 132.76 (C), 130.34 (CH), 127.10 (CH), 126.80 (CH), 123.01 (CH), 33.87 (CH_2_), 31.74 (CH_2_), 29.47 (CH_2_), 29.45 (CH_2_), 29.38 (CH_2_), 29.28 (CH_2_), 29.15 (CH_2_), 29.08 (CH_2_), 28.79 (CH_2_), 24.69 (CH_2_), 22.55 (CH_2_), 14.40 (CH_3_).

### 2.2 Computational details

The molecular structures of acid dimers were fully optimized without geometrical constraint via GAUSSIAN 09 program [49] and were visualized using GaussView 05 [50]. Following the successful optimization, the frequency calculation was conducted to substantiate the convergence nature of the dimers and real values were predicted for all the frequencies. On the other hand, the molecular electrostatic potential (MEP) surfaces and frontier molecular orbitals were obtained using the optimization's supplemental check files (.chk), All the computations were accomplished by density functional theory (DFT) using B3LYP method [51,52] while 6-311G was used as the basis set.

## 3 Results and discussion

### 3.1 Chemistry

Scheme 1 provides an illustration of the synthetic pathway leading to the target molecules. Concisely, compounds **I***n* and **II***n* were prepared with a typical acyl chloride-phenol condensation procedure. Quaternary ammonium salt derived from acyl chloride was formed in situ by addition of triethylamine to an anhydrous solution of acid chloride in presence of catalytic amount of dimethylaminopyridine (DMAP). Then the activated acyl groups (namely, hexanoyl, decanoyl or hexadecanoyl) were allowed to react with either 3- or 4-hydroxybenzoic acid to afford 3- or 4- alkanoyloxybenzoic acids respectively. The reaction time ranged from 20 to 24 h at room temperature, and the yields were between 75 and 86% and recrystallization was used to purify all products.

The target alkanoyloxybenzoic acids were primarily characterized for the presence of the ester functional group and for the terminal alkyl chain in their structures. In the proton NMR spectra, chemical shifts for the aliphatic protons of the terminal alkyl chain were assigned in the shielding region (*δ* = 0.85–3.18 ppm). Chemical shifts for the aromatic protons were observed in the low fields with signals at *δ* = 7.24～7.99 ppm corresponding to the four protons of the aromatic ring. In the ^13^C NMR spectra, the terminal alkyl chain saturated carbons of (*δ*) appeared in high fields at chemical shifts *δ* = 14.09～45.51 ppm, while benzene-ring unsaturated carbons appeared in low fields at *δ* = 122.48～154.25 ppm. Furthermore, the chemical shifts of carbonyl carbon atom either in carboxy or ester functional groups were observed at lower fields at *δ* = 179.91～ 167.12 ppm due to the substantial de-shielding effects of carbonyl oxygen and the magnetic anisotropy effect of the adjacent aromatic ring. As an exception, compound **I***7* showed a duplicate number for the saturated carbons of the terminal alkyl chain, which indicate that acylation take place for both phenolic and carboxy group affording acid anhydride with high thermal stability.

### 3.2 Mesomorphic behaviours of acid dimers

**Fig. 1:**
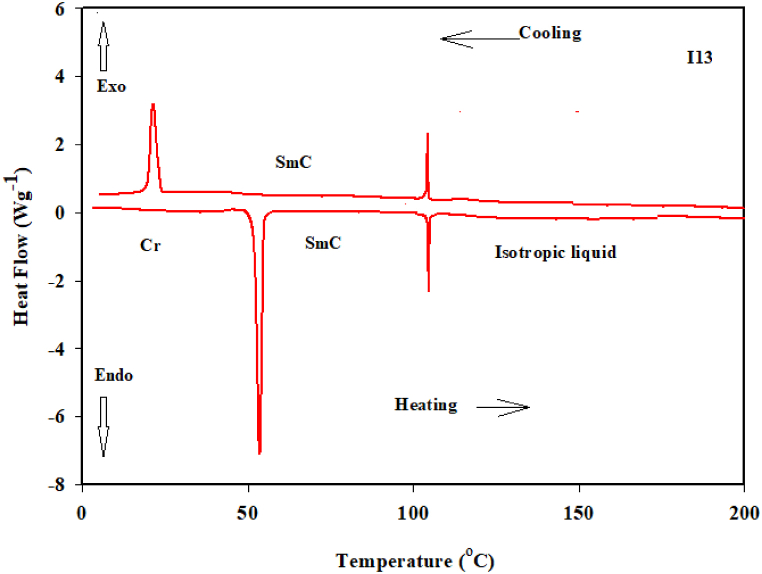
DSC thermograms of **I***13* acid dimer from the second heating/cooling cycle, at a rate 5 °C min^−1^.

**Fig. 2:**
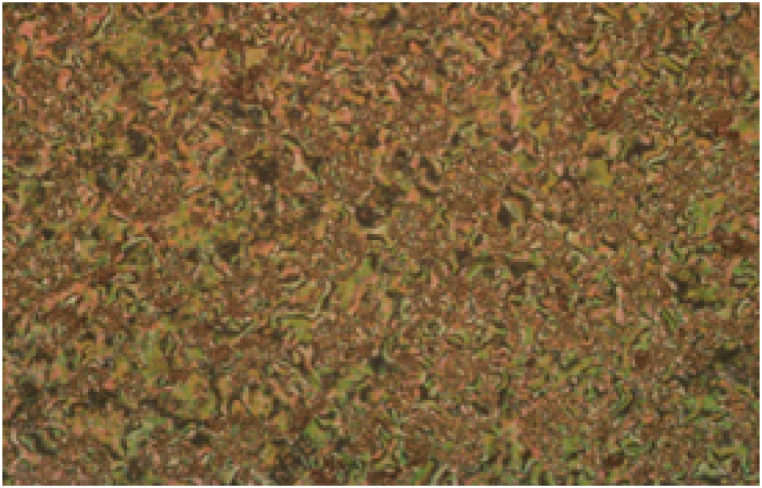
SmC texture of **I***13* dimeric complex under POM upon heating at 91 °C.

**Fig. 3:**
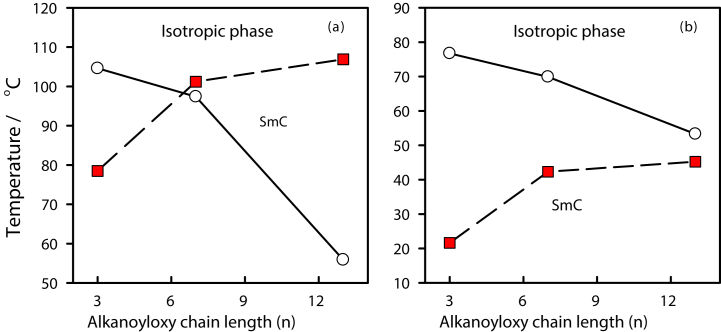
Effect of the length of alkanoyloxy chain on the mesophase behavior of the supramolecular dimers, (a) **I***n* and **(b) II***n*. (Open circle) melting point, (b) (solid square) SmC stability.

The two groups of 3- (or 4-)-*n*-alkanoyloxy benzoic acids (**I***n* and **II***n*) were synthesized, and the possible supramolecular binary complexes that formed from each dimer of the same acid, were thoroughly described. DSC and POM were utilized to evaluate the mesomorphic characteristics of the resultant mixtures. Fig. 1 displays the DSC thermogram of **I***13* dimer as representative example. Fig. 2 depicts typical textures of the mesophases under POM. Mesomorphic transition temperatures and their related enthalpy for each supramolecular H-bonded acid dimer complex are elaborated in Table 1. To investigate the impact of the length of terminal alkanoyloxy chain on the mesomorphic behavior of both prepared dimeric groups, Fig. 3a and b plots the phase transition temperatures as a function of the alkanoyloxy chain length (n), as determined by DSC experiments, which varies among 3, 7 and 13 carbons. Fig. 3a and b and Table 1 demonstrate that the melting points for all prepared dimers shift irregularly as the alkanoyloxy chain length increases, as usual in LCs systems [17,18]. Furthermore, depends on the terminal side chain lengths and their positions, all complexes were shown to exhibit monomorphic SmC phase in either an enantiotropic or monotropic approach. For complexes **I***3*, **I***7*, and **I***13*, respectively, the designed supramolecular hydrogen bonded complexes (**I***n*) exhibit mesophases with thermal stabilities of 78.5, 101.2, and 106.9 °C. Additionally, as n increases, the observed smectic C (SmC) phase becomes significantly more stable. The formed SmC for **I***3* symmetrical dimer appears monotropically and has the least thermal stability in group **I***n* (78.5 °C). The mesophase range and stability of the complex **I***7*'s SmC phase are around 3.8 and 101.2 °C, respectively. The highest thermal stability and smectogenic range, which are about 106 and 51.0 °C, respectively, are displayed by the longest dimeric complex **I***13*. These findings are in consistent with earlier publications [36]. All dimeric compounds in the second series **II***n* (Fig. 3b) show monotropic characteristics. Additionally, for **II***3*, **II***7*, and **II***13*, respectively, their SmC stabilities increase in order to 21.6, 42.3, and 45.2 °C. The dimer **II***13* in the series **II***n* has the maximum thermal stability, while the supramolecular complex in the complex **II***3* exhibits the lowest smectogenic stability. The produced dimers have a preferred molecular configuration, as detected from all mesomorphic behavior studies.

**Table 1:** The measured phase transition temperatures (°C), enthalpy (kJ/mol), and normalized entropy for the **I***n* and **II***n* series.

System	*T*_Cr-SmC_	*ΔH*_Cr-SmC_	*T*_SmC_-_I_	*ΔH*_SmC-I_	*ΔS/R*
**I***3*	104.6	49.65	78.5^a^	3.4	1.16
**I***7*	97.4	40.21	101.2	2.3	0.74
**I***13*	55.9	41.72	106.9	2.5	0.79
**II***3*	76.7	39.66	21.6^a^	1.3	0.53
**II***7*	69.9	45.12	42.3^a^	1.9	0.72
**II***13*	53.3	43.99	45.2^a^	2.2	0.83

Cr-SmC = crystal - smectic C phase transition.

SmC-I = smectic C - isotropic liquid phase transition.

a Monotropic phase appear on cooling onlyR is the universal gas constant.

For all dimers (**I***n* **and II***n*), the normalized entropy changes of transitions (***ΔS/R***) were estimated, and the results are listed in Table 1. With respect to group **I***n*, the entropy changes (***ΔS/R***) decreases as the length of alkanoyloxy chain increased from *n* = **3** to **7** carbons slightly increases for *n* = **13**. The varied stability of the created mesophases may be responsible for this inconsistent relationship. It can be noted from Table 1 that the entropy changes (***ΔS/R***) for group **II***n* demonstrates an increment values with the increment of the alkanoyloxy side chain. Because of the establishment of a broad range of SmC phase, the degree of lateral associations between molecules is increasing along with the overall length of the molecule. Additionally, the weak conjugative interactions between the mesogenic cores of the examined dimeric complexes are responsible for the relatively low values of entropy change reported.

### 3.3 Optical properties

**Fig. 4:**
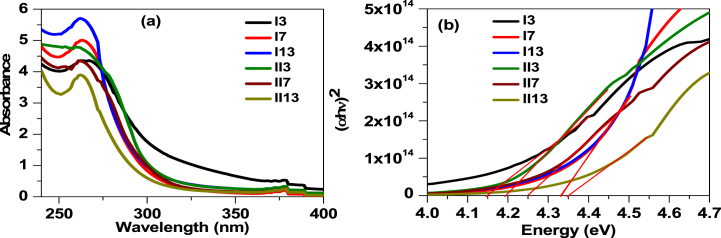
The absorption spectra of liquid crystalline films of (a) 4-alkanoyloxy benzoic acid and (b) 3-alkanoyloxy benzoic acid.

Finding uses for liquid crystalline materials in optoelectronic devices requires an understanding of their optical characteristics. For this purpose, we have investigated the emission and absorption spectra of the prepared LCs. Fig. 4a shows the absorption spectra of both *p*-alkanoyloxy (**I***n*) and *m*-alkanoyloxy (**II***n*) benzoic acid. The absorbance band around λ = 265 nm was assigned to the *C*-band for compound **II***3* [53,54]. As the length of the alkyl side chain increases, peak slightly blue shifted to 264 nm (for **II***7*) and 265 nm (for **II***13*). The blue shift in absorption spectra originated due to steric hindrance of the alkyl side chain [55]. The absorption spectra of meta samples were found similar to the para samples. The energy band gap of liquid crystalline 4-alkoyloxy benzoic acid and 3-alkanoyloxy benzoic acid were evaluated through Tauc plot shown in Fig. 4b. According to Tauc relation [56,57] ${\left(\alpha hv\right)}^{2}=A\left(E-E_{g}\right)$, the intercept of the tangent on energy axis provides the band gap. The band gap of **I***3* sample was found to be 4.14 eV and noted to be increased to 4.33 eV for **I***13* sample. The bandgap of **II***n* samples were slightly higher than **I***n* samples with Eg = 4.19 eV, 4.25 eV and 4.35 eV for **II***3*, **II***7* and **II1***3*, respectively.

**Fig. 5:**
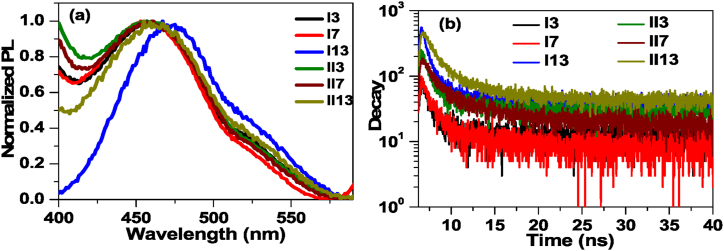
(a) Steady state and (b) time resolved fluorescence spectra of mesomorphic alkoyloxy benzoic acid.

The normalized photoluminescence (PL) spectra of liquid crystalline alkanoyloxy benzoic acids were recorded by exciting the sample with 320 nm (Fig. 5a and b). The materials exhibit broad emission spectra in the range of 410–575 nm with peak emission ⁓ 460 nm. As the length of the side chain increased, a red shift was observed in the emission spectra. In addition, a minor red shift was observed in the emission peak of **II**n compared to **I**n., with peak emission at 472 nm for **II***13*. The fluorescence decay spectra shown in Fig. 5b was recorded to study the effect of side length of terminal chain on life time of excited charge carriers. It was found that the decay spectra are well fitted with an exponential decay function: I(t)=A+Be−tτ, where A and B are fitting constants and τ is charge carrier lifetime. The evaluated fluorescence decay parameters are presented in Table 2. The lifetime was evaluated to be 156 ps, 232 ps and 1.981 ns for the samples **I***3*, **I***7* and **I***13*, respectively. Results show that as alkyl side chain length rises, so does the lifetime. Further, the life time for the samples having functional group at m-position (**II***n*) noted to be higher than the samples having functional group at p-position (**I***n*). Again, the lifetime for m-positional samples were also noted to be increasing with lengthen of side chain length with lifetime of 489 ps, 519 ps, and 855 ps, for the samples **I***I3*, **II***7* and **II***13*, respectively.

**Table 2:** Energy band gap and life time of liquid crystalline alkoyloxy benzoic acid samples.

Sample	I*3*	I*7*	I*13*	II*3*	II*7*	II*13*
**Eg (eV)**	4.14	4.33	4.33	4.19	4.25	4.35
**ṭ (ps)**	156	232	1981	489	519	855

### 3.4 DFT theoretical studies

#### 3.4.1 Relative stability

The compounds stability could be influenced by the substituent position The result presented in Table 3, affirms the *para*-alkanoyl derivatives (**I***n*) of the acid dimer to be of higher stability relative t the corresponding *meta*-alkanoyl counterpart (**II***n*) by 3 kcal/mol. The orientation of the ipso oxygen of the ester substituent in the acid would affect its electron donating ability, which in principle thus, it would be more active at para position than the meta and providing the greater stability to **I*n*** series over that of **II***n* via resonance [50,51]. In addition, steric hindrance is minimal at para position, and this could have aided the stability of **I***n* dimers over the **II***n* counterparts [58]. Another important factor that could have enhanced the stability of **I***n* derivatives might be the interatomic hydrogen bonding interaction between the acids in the dimer. The slightly shorter OH---OC interatomic distance recorded for the para derivatives (Table 3) suggests greater hydrogen bonding interaction, which in return aided their stability over the *meta*-alkanoyl analogues. Furthermore, the greater deviation of OH⋯O bond angle of **II***n* dimers from the linearity (180°) as highlighted in Table 3 attests to their weaker hydrogen bonding interaction compared to the corresponding components in series [59,60]. The theoretical revelations about the greater stability of the **I***n* series over the **II***n* counterparts are in consistent with the observations from the thermal stability of the mesophases revealed by the phase transition experiment.

**Table 3:** Relative energy and selected structural parameters calculated for the acid dimers **I***n* and **II***n* at B3LYP/6-311G level.

Compound	Relative energy (kcal/mol)		OH---OC Inter atomic distance (Å)		OH⋯O Bond angle (^o^)	
**n**	**In**	**IIn**	**In**	**IIn**	**In**	**IIn**
**3**	0	2.82	1.607	1.616	176.3	175.4
**7**	0	2.79	1.609	1.617	176.2	175.3
**13**	0	2.71	1.610	1.617	176.2	175.3

#### 3.4.2 Reactivity parameters

Both HOMO-LUMO energies and MEP are important for the understanding of the absorption and emission spectra of liquid crystals. These properties allow for the interpretation of the electronic structure and vibrational modes of molecules, which is critical for the development of liquid crystal materials with novel optical and electronic characteristics. HOMO-LUMO energies refer to the energy difference between the highest occupied molecular orbital (HOMO) and the lowest unoccupied molecular orbital (LUMO). This energy difference determines the energy required for an electron to transition from the HOMO to the LUMO, which is related to the absorption spectrum. The absorption spectrum of a liquid crystal provides information about the energy required to excite the molecules, which determines the colors that are absorbed and transmitted by the material.

Moreover, the HOMO-LUMO energy gap is a crucial parameter in accessing the reactivity of chemical compounds. Other indicators for chemical reactivity are the electron affinity (EA) and ionization potential (I.P) [61,62]. To evaluate the positional effect of the ester moiety together with the increased length of its alkyl part on the reactivity of acid dimers resulting from the hydrogen bonding interaction, the calculation was carried out while the computed reactivity parameters are listed in Table 4. The result reveals that the HOMO and LUMO energy parameters are being influenced by the position of ester substituent. Both HOMO and LUMO energy levels of the *para*-alkanoyl derivatives (**I***n* series) were predicted to be lower than that of the corresponding *meta*-alkanoyl counterparts (**II***n* series). At the end, lower resultant HOMO-LUMO energy gaps (ΔE) were obtained for the **II***n* members. The smaller energy gap suggests that the **II***n* would be more reactive but less stable than the **I***n* analogues [51] which is in consistent with the stability of earlier predicted from the relative energy. On the other hand, the reactivity parameters seem to be less sensitive to the size of terminal alkyl whose similar values were calculated for the respective HOMO and LUMO energy levels in both series of the acid dimer. With respect to the polarity of acid dimer, it could be seen that it is affected by the ester position and the length of terminal alkyl as revealed by the calculated dipole moment and isotropic polarizability highlighted in Table 4. The higher value obtained for the dipole moment of the **I***n* series can be attributed to their greater polarity over the **II***n* counterparts and this is further corroborated by the higher isotropic polarizability computed for them [2]. The observed decrease in the dipole moment for both **I***n* and **II***n* with increasing the length of the dimer terminal alkanoyl, is consistent with the reports for the hydrogen bonded dimers having the magnitude of their dipole moment decreasing with the increase in size of the system [2,63,64].

**Table 4:** Reactivity and polarity parameters of the acid dimers calculated at the B3LYP/6-311G level.

Compound	E_HOMO_ (eV)		E_LUMO_ (eV)		ΔE (eV)		Dipole moment (Debye)		I·P (eV)		E.A (eV)		Isotropic polarizability (Bohr**3)	
**n**	**I***n*	**II***n*	**I***n*	**II***n*	**I*n***	**II***n*	**I***n*	**II***n*	**I***n*	**II***n*	**I***n*	**II***n*	**I***n*	**II***n*
**3**	−7.156	−7.062	−1.988	−1.951	5.168	5.111	2.8528	0.2765	7.156	7.062	1.988	1.951	333.35	320.86
**7**	−7.154	−7.059	−1.984	−1.949	5.170	5.110	2.772	0.1916	7.154	7.059	1.984	1.949	428.66	415.17
**13**	−7.153	−7.058	−1.984	−1.948	5.169	5.110	2.7304	0.1702	7.153	7.058	1.984	1.948	570.26	556.15

One important characteristic of liquid crystals is their isotropic polarizability, which refers to their ability to respond to an external electric field by rearranging their molecular structure. Isotropic polarizability is directly associated to the capability of liquid crystals to be manipulated and controlled in various applications such as sensors, displays, and optical devices. It is a measure of how easily the liquid crystal molecules can align themselves in response to an applied electric field. Controlling the isotropic polarizability of liquid crystals is essential for the design and optimization of liquid crystal devices. By selecting or synthesizing liquid crystal materials with specific polarizability characteristics, it is possible to tailor their response to external stimuli and optimize their performance in various applications. Researchers continue to explore novel liquid crystal compounds with enhanced isotropic polarizabilities to further advance the field of liquid crystal technology. Liquid crystal molecules have an elongated shape, with a rod-like structure. These molecules are typically polar, meaning they have a positive and negative end. As response to applying an electric field, the positive and negative ends of the liquid crystal molecules tend to align themselves parallel to the field. This alignment is what allows the liquid crystal to change its optical properties, such as the orientation of polarized light passing through it. The isotropic polarizability of liquid crystals is determined by several factors, including the shape and size of the molecules, as well as the nature of the intermolecular forces between them. Smaller liquid crystal molecules with a higher aspect ratio have higher isotropic polarizabilities, meaning they can more effectively respond to an external electric field. Additionally, the presence of side chains or substituents on the liquid crystal molecules can also influence their polarizability. As chain length increases, polarizability also rise; nevertheless, **In** series are more polarizable than **IIn** series. According to Table 4, the mesophase stability improves as the polarizability increases. Smectic phase's presence can be explained in terms of the increase in polarity and polarizability with lengthening of the alkoxy chain. As the alkoxy chain length rises, the chains aggregate more frequently in the ordered smectic phase [65].

**Fig. 6:**
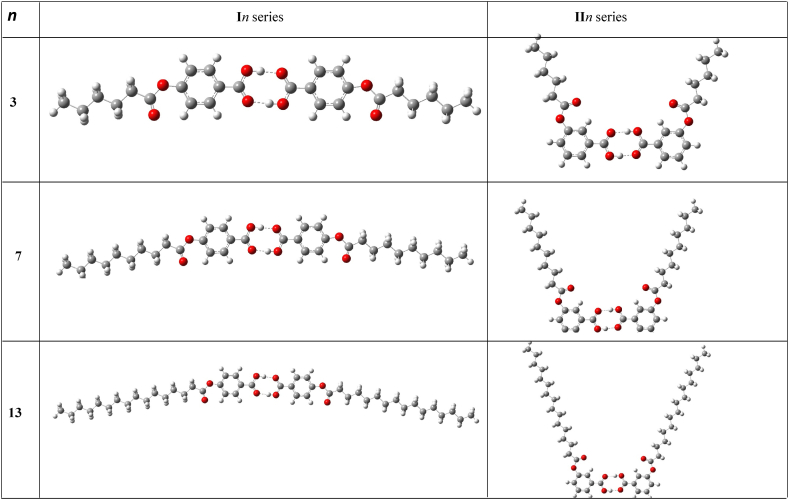
Structures of the **I***n* and **II***n* acid dimers calculated at B3LYP/6-311G.

**Fig. 7:**
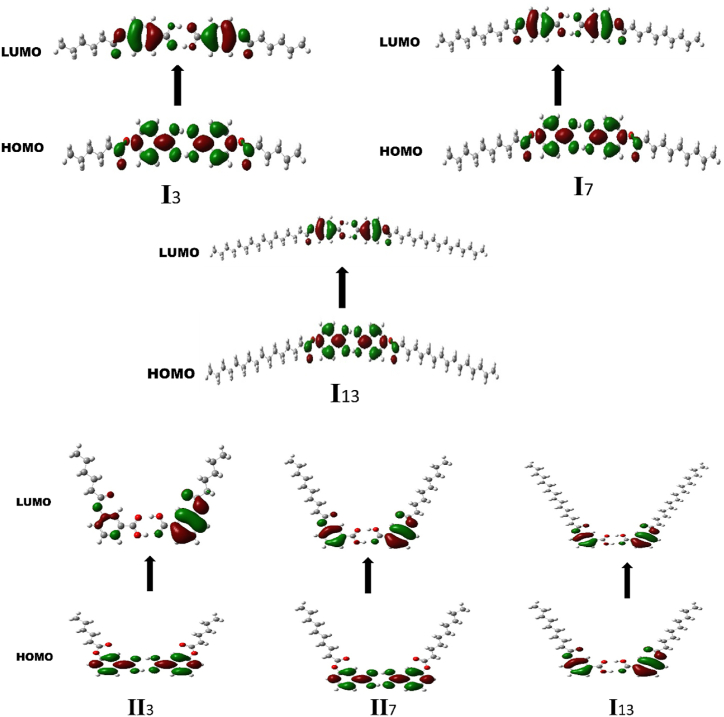
Frontier molecular orbitals calculated at B3LYP/6-311G for **I***n* and **II***n*.

**Fig. 8:**
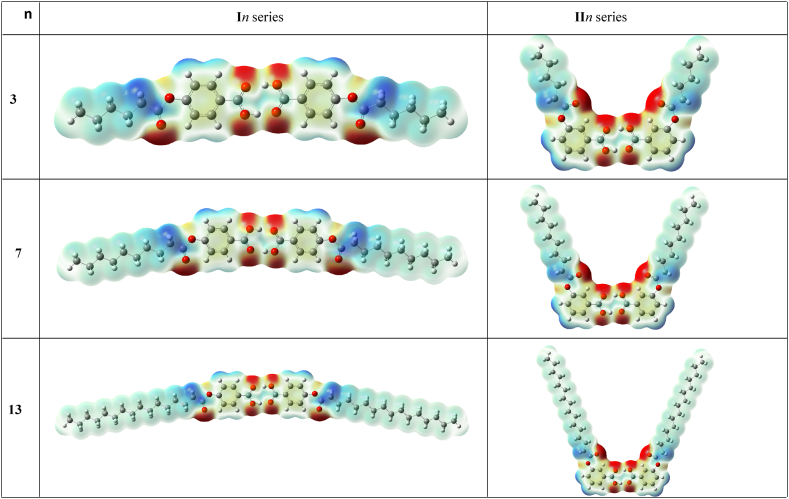
Molecular electrostatic potentials of the **I***n* and **II***n* acid dimers determined at isovalue of 0.02.

The study of frontier molecular orbitals, the representative models of which are shown in Fig. 6, Fig. 7, for both dimer of **I***n* and **II***n*, respectively, exhibits some similarities in the orbitals distribution at the HOMO level, as they have their electron clouds being steadily distributed over only the carbon atoms of the phenyl rings. In addition, similar appreciable electron clouds distribution upon the carbon and oxygen atoms of C=O and the alkoxy oxygen of the carboxylic moiety was predicted for the two acid dimers (**I***n* and **II***n*). However, their HOMOs differ at alkanoate part, as noticeable electron densities were obtained for the carbon and oxygen of the C=O of the alkanoate of **I***n* series, however, the **II***n* counterparts found to be electron deficient. In the case of the LUMO, electron clouds distribution significantly covered both carbon atoms and the π system of phenyl ring for the **I***n* and **II***n* dimers. Moreover, electron clouds were predicted to be fairly spread over the carbonyl and alkoxy oxygen atoms of both alkanoate and carboxylic moieties of **I***n* members, whereas the electron deficiency was noted for the –COOH group of the **II***n* derivatives. Another computed parameter worth mentioning is the molecular electrostatic potential (MEP). It is a measure of the electrostatic potential energy distribution of electrons in a molecule. It is related to the electronic density distribution around the molecule and provides information about the charge distribution on the molecule Thus, MEP normally reveals the extent of electron density for a particular region (Fig. 8). The red cloud over the carbonyl and alkanoyl oxygen atoms of the carboxylic moiety and carbonyl oxygen of the terminal alkanoate of both **I***n* and **II*n*** dimers, portrays these regions to be of high electron density but with low electrostatic potential. However, the blue cloud over the region of alkanoate carbonyl carbon and its immediate methylene for the two acid dimers signifies high electrostatic potential with low electron density [[56], [57], [58]].

#### 3.4.3 Energy

Being an extensive property, the magnitude of energy is always dependent on the size of the system. This assertion is justified by the trend of increasing value of zero-point energy, thermal energy, and the thermodynamic variables with the size of the dimers as computed in Table 5. This observation assents to the reports on the energy of systems being directly related to their sizes [66,67]. In addition, the trend of increasing calculated thermal energy with the size of the system is consistent with the increasing thermal stabilities of mesophases for both **I***n* and **II***n* series with the length of alkanoyloxy chain, as revealed by the smectic C to isotropic liquid phase transition temperatures (***T***_SmC_-_I_). Theoretically, as the length of the terminal chain (n) increases, so do the estimated to enthalpy changes for molecules. Additionally, for both series, predicted entropy change rises.

**Table 5:** Zero-point energy, thermal energy and thermodynamic variables in kcalmol^−1^ for the acid dimers **I***n* and **II***n* calculated at the B3LYP/6-311G level.

Compound	ZPE (kcal/mol)		Thermal (kcal/mol)		Enthalpy (kcal/mol)		Gibbs (kcal/mol)		Entropy (cal/mol K)	
**n**	**I***n*	**II***n*	**I***n*	**II***n*	**I***n*	**II***n*	**I***n*	**II***n*	**I***n*	**II***n*
**3**	340.662	340.644	363.158	363.112	363.750	363.704	291.528	291.246	242.235	243.025
**7**	483.987	483.955	513.287	513.231	513.879	513.824	424.927	424.314	298.347	300.217
**13**	699.002	698.913	738.489	738.404	739.081	738.996	624.885	623.905	383.015	386.019

## 4 Conclusion

In conclusions, six new symmetrical 3- (or 4-)-*n*-alkanoyloxy benzoic acid liquid crystals were synthesized and investigated by experimental and theoretical tools. The results revealed that all dimers are monomorphic exhibiting purely SmC phase. The theoretical results fairly agreed with experimental data on the mesomorphic behavior and further revealed the **I***n* series to be more stable but less reactive than the **II***n* counterparts. Moreover, the predicted reactivity parameters suggested that the position of ester substituent appreciably affected the electronic properties of the benzoic acids under studied. Optical bandgap was found to be increases with the increase of alkanoyl side chain length and meta-derivatives have slightly higher bandgap as compared to the para-derivatives. Moreover, the fluorescence lifetime was found to be increases with the increase of alkanoyl side chain length, and meta-derivatives have slightly longer lifetime as compared to the para-derivatives.

## Declarations

### Author contribution statement

Mohd Taukeer Khan: wrote the paper; contributed reagents, materials, analysis tools or data; performed the experiments; conceived and designed the experiments.

Saheed A. Popoola; Muna S. Khushaim; Mariusz Jaremko: performed the experiments; analyzed and interpreted the data; wrote the paper.

Magdi M. Naoum: conceived and designed the experiments; analyzed and interpreted the data; contributed reagents, materials, analysis tools or data; wrote the paper.

Mohamed A. El-Atawy: wrote the paper; contributed reagents, materials, analysis tools or data; analyzed and interpreted the data; performed the experiments; conceived and designed the experiments.

Hoda A. Ahmed: conceived and designed the experiments; performed the experiments; contributed reagents, materials, analysis tools or data; wrote the paper.

Abdul-Hamid Emwas, Fowzia S. Alamro: performed the experiments; contributed reagents, materials, analysis tools or data; wrote the paper.

### Data availability statement

Data included in article/supp. Material/referenced in article.

## Declaration of competing interest

The authors declare that they have no known competing financial interests or personal relationships that could have appeared to influence the work reported in this paper.
