# Supplementary material for: First mesomorphic and DFT characterizations for 3- (or 4-) n-alkanoyloxy benzoic acids and their optical applications
Source: Heliyon. 2023 Aug 24;9(9):e19384. doi: 10.1016/j.heliyon.2023.e19384 (PMC10472244; doi:10.1016/j.heliyon.2023.e19384)
Supplement: Multimedia component 1 [file mmc1.docx]

**Supplementary data**

**Mesomorphic and DFT Characterizations for supramolecular Hydrogen bonding of 3- (or 4-) n-alkanoyloxy benzoic acids and their optical applications**

**Mohamed A. El-Atawy^1,2^, Mohd Taukeer Khan^3^, Saheed A. Popoola^4^,** **Muna S. Khushaim^5,6^, Mariusz Jaremko^7^, Abdul-Hamid Emwas ^8^, Fowzia S. Alamro ^9^ , Magdi M. Naoum^10^ and** **Hoda A. Ahmed ^10,^***

1. Chemistry Department, Faculty of Science, Alexandria University, P.O. Box 426, Ibrahemia, Alexandria 21321, Egypt; mohamed.elatawi@alexu.edu.eg
2. Chemistry Department, College of Sciences, Taibah University, Yanbu 30799, Saudi Arabia
3. Department of Physics, Faculty of Science, Islamic University of Madinah,

Al-Madinah Al-Munawwarah 42351, Saudi Arabia; [khanmtk@iu.edu.sa](mailto:khanmtk@iu.edu.sa)

1. Chemistry Department, Faculty of Science, Islamic University of Madinah, Madinah Saudi Arabia; [abiodun@iu.edu.sa](mailto:abiodun@iu.edu.sa)
2. Department of Physics, Faculty of Science, Taibah University, P.O. Box 30002,

Al-Madina 41447, Saudi Arabia; mkhushaim@taibahu.edu.sa

1. Nanotechonolgy Center, Taibah University, P.O. Box 30002, Al-Madina 41447, Saudi Arabia
2. Biological and Environmental Sciences & Engineering Division (BESE), King Abdullah University of Science and Technology (KAUST), Thuwal, 23955-6900, Saudi Arabia ; [Mariusz.jaremko@kaust.edu.sa](mailto:Mariusz.jaremko@kaust.edu.sa)
3. Core Labs., King Abdullah University of Science and Technology, Thuwal 23955-6900, Saudi Arabia; [abdelhamid.emwas@kaust.edu.sa](mailto:abdelhamid.emwas@kaust.edu.sa)
4. Department of Chemistry, College of Science, Princess Nourah bint Abdulrahman University, P.O. Box 84428, Riyadh 11671, Saudi Arabia; [fsalamro@pnu.edu.sa](mailto:fsalamro@pnu.edu.sa)
5. Department of Chemistry, Faculty of Science, Cairo University, Giza 12613, Egypt; [ahoda@sci.cu.edu.eg](mailto:ahoda@sci.cu.edu.eg) (HAA); [magdinaoum@yahoo.co.uk](mailto:magdinaoum@yahoo.co.uk) (MMN)

* Correspondence: [ahoda@sci.cu.edu.eg](mailto:ahoda@sci.cu.edu.eg) (H.A.A.)

Figure S1.^1^H-NMR of 3-(palmitoyloxy)benzoic acid

Figure S2.^13^C-NMR of 3-(palmitoyloxy)benzoic acid

Figure S3.^1^HNMR of 3-(decanoyloxy)benzoic acid

Figure S4.^13^C-NMR of 3-(decanoyloxy)benzoic acid


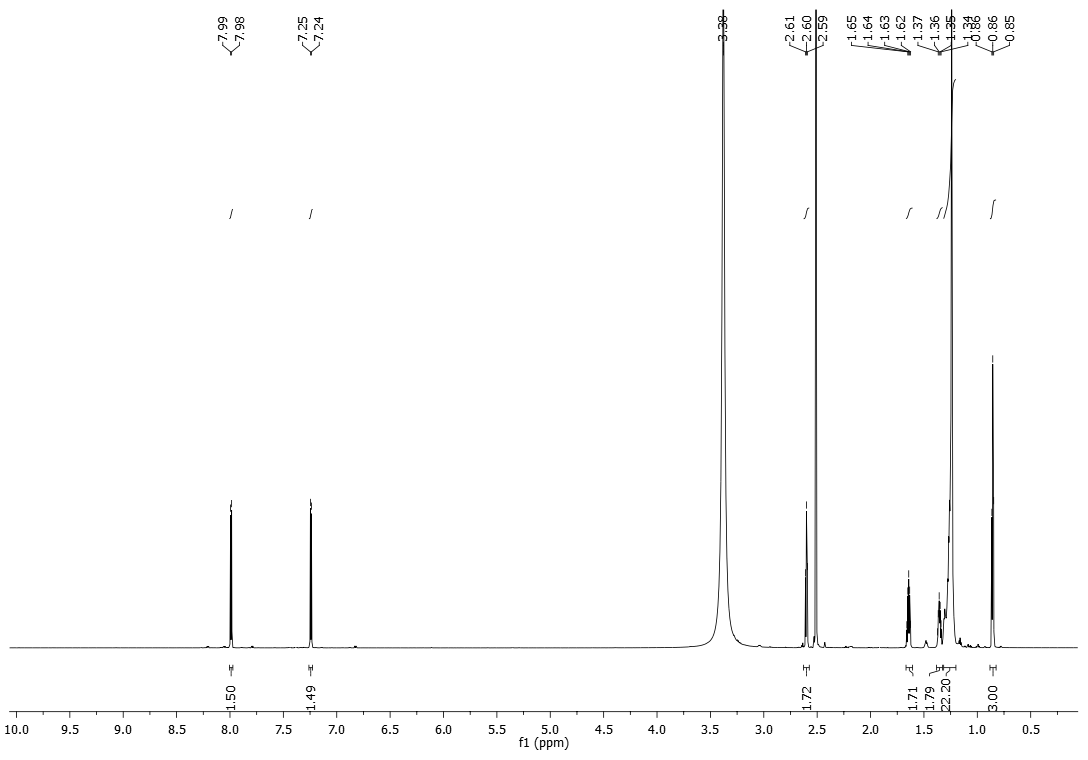

Figure S5.^1^H-NMR of 4-(palmitoyloxy)benzoic acid

Figure S6.^13^C-NMR of 4-(palmitoyloxy)benzoic acid


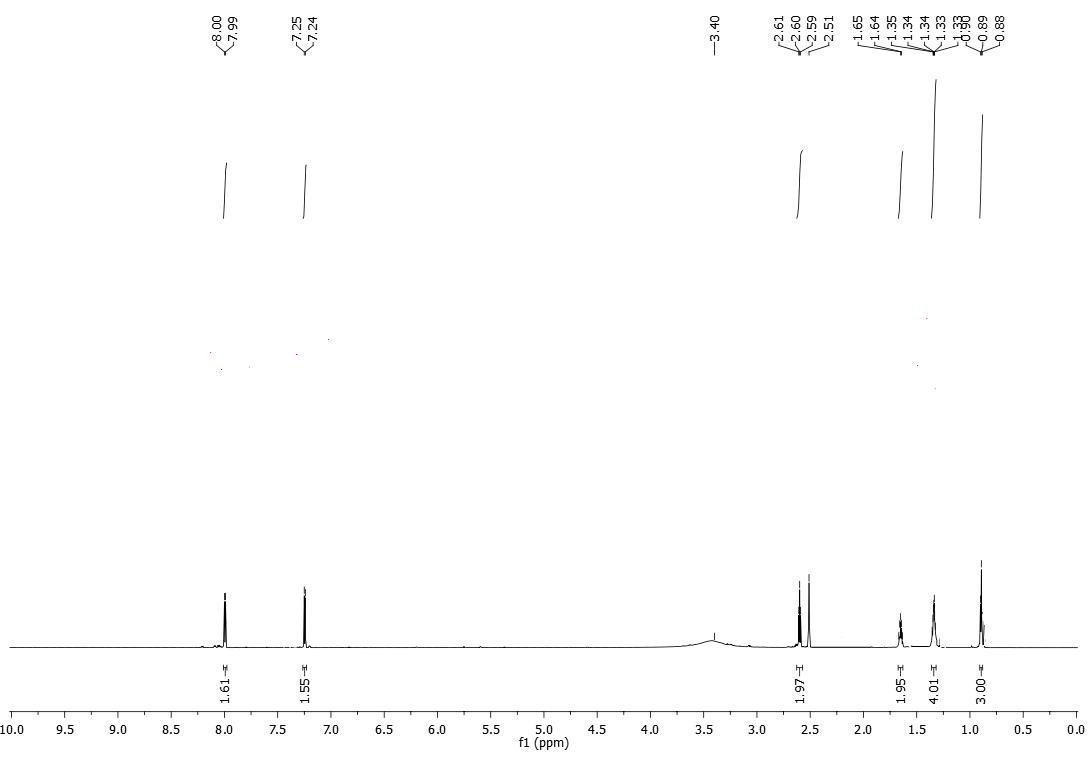

Figure S7.^1^H-NMR of 4-(hexanoyloxy)benzoic acid


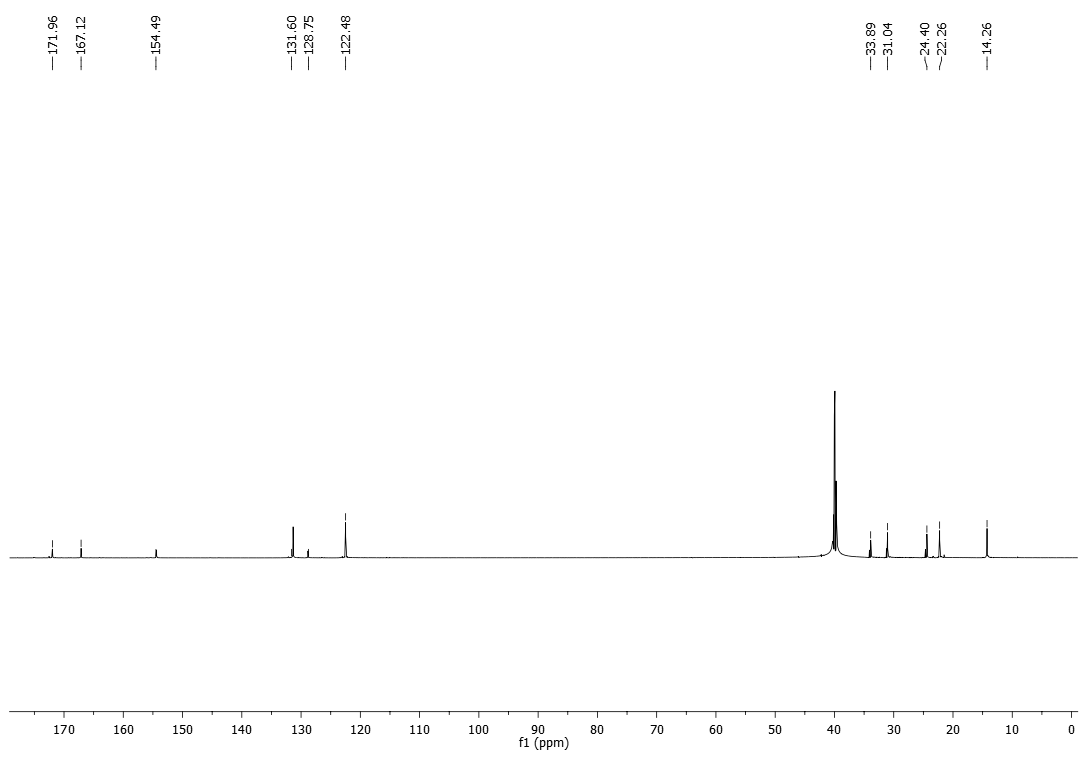

Figure S8.^13^C-NMR of 4-(hexanoyloxy)benzoic acid

Figure S9.1H-NMR of decanoic 4-(decanoyloxy)benzoic anhydride

Figure S10.^13^C-NMR of decanoic 4-(decanoyloxy)benzoic anhydride
